# Editorial: The Developments of Hybrid Surgical Strategies for Congenital Heart Disease

**DOI:** 10.3389/fsurg.2017.00055

**Published:** 2017-09-22

**Authors:** Filippo Rapetto, Damien Kenny, Massimo Caputo

**Affiliations:** ^1^Department of Cardiac Surgery, Bristol Heart Institute, University of Bristol, Bristol, United Kingdom; ^2^Department of Cardiac Surgery, Bristol Royal Hospital for Children, University of Bristol, Bristol, United Kingdom; ^3^Department of Paediatric Cardiology, Rush University Medical Center, Chicago, IL, United States

**Keywords:** cardiac surgery, hybrid surgery, interventional cardiology, congenital heart disease, surgical risk

**Editorial on the Research Topic**

**The Developments of Hybrid Surgical Strategies for Congenital Heart Disease**

Over the last few decades, we have witnessed a significant improvement in the outcomes of congenital cardiac surgery and a huge expansion in interventional cardiology techniques for the treatment of congenital heart disease. Nevertheless, a considerable number of patients fall into a gray area in which on the one hand conventional surgery has an unsatisfactory risk, and on the other hand an interventional cardiology procedure is not feasible because of technical limitations.

A hybrid operation is defined as a procedure carried out by means of a combination of surgical and interventional strategies. If we consider the abovementioned gray area, a hybrid operation can be seen as a surgical procedure whose unsafe steps are dealt with interventional techniques; similarly, we could define it as an interventional procedure whose technical issues are overcome or reduced by one or more surgical steps.

Having broadly defined hybrid surgery, some more detailed definitions are needed to better understand the rationale behind it.

From a surgical perspective, duration of cardiopulmonary bypass and cardioplegic arrest of the heart are two of the main determinants of perioperative risk, which is directly correlated with them (1). This general concept applies to both pediatric (and especially neonatal) patients and adult patients with congenital heart disease. In this context, a hybrid approach can have an impact on surgical risk by either avoiding or reducing cardiopulmonary bypass and cardioplegic arrest times and therefore by modifying variables such as surgical trauma, inflammation, and postoperative systolic dysfunction. Second, hybrid surgery expands the available options when facing reoperations, which are becoming more and more common in adult patients with congenital heart disease because of increased life expectancy in the current era. Despite the emergence of alternative accesses (2, 3), the vast majority of conventional cardiac reoperations are still performed through redo median sternotomy. Even though complications during reentry are significantly reduced nowadays, this access itself has proven to be a risk factor for early adverse events, especially in patients who need more than one redo sternotomy over time (4, 5). Hybrid surgery can, in selected cases, allow for a less invasive access when the expected risk of reentry is high.

From an interventional cardiology perspective, technical issues are related to limited size range of the available devices and awkward catheter manipulation in the context of the complex anatomy typical of congenital heart disease. Hence, the surgical portion of a hybrid operation aims at overcoming size limitations and providing a more direct access for catheter-based procedures.

Being able to limit surgical risk has not only therapeutic implications but it also affects preoperative assessment process and, potentially, surgical indication. Therefore, the availability of a hybrid option broadens the spectrum of patients who can be treated successfully, including extremely high-risk individuals.

The purpose of this research topic is providing an overview of the most important clinical problems that can be addressed with a hybrid approach in pediatric and adult congenital heart disease. It consists of four papers exploring different applications of hybrid surgery in this field.

In their review on hybrid pulmonary valve replacement after surgery for tetralogy of Fallot, Suleiman and colleagues reported current outcomes of surgical and percutaneous pulmonary valve replacement, highlighting drawbacks for both approaches. They also illustrated the potential advantages of hybrid pulmonary valve replacement according to experiences from the Bristol Heart Institute and the Rush University Medical Center, providing technical insights in terms of operative strategy and reporting published data supporting their practice (Suleiman et al.).

Rapetto and coworkers published a short series of three cases of adult patients with congenital heart disease that were managed successfully with a hybrid surgical approach. All patients had undergone previous cardiac operations for different reasons; they were then deemed unsuitable for conventional reoperative surgery requiring cardiopulmonary bypass and cardioplegic arrest due to comorbidities, impaired ventricular function or both (Rapetto et al.).

Gupta and Amin reviewed the most popular hybrid congenital heart operations, specifically focusing on stage I Norwood procedure, ventricular septal defect closure, pulmonary valve replacement, and aortic balloon valvuloplasty. For each procedure, they briefly summarized the rationale for patient selection, technical aspects, and clinical outcomes, and also pointing out that the collaboration among different specialists is the necessary premise for satisfactory results (Gupta and Amin).

Finally, McLennan and colleagues reported a case of an infant with combination of aortic coarctation and aortic valvar stenosis, treated by surgical aortic coarctation repair and hybrid aortic balloon valvuloplasty through carotid artery cutdown. The two steps were performed as a combined procedure in a hybrid operating theater, with coarctation repair immediately followed by valvuloplasty. The authors also reviewed previously published outcomes of surgical and interventional treatments for these conditions, highlighting the excellent surgical results of conventional surgery for aortic coarctation but also pointing out the drawbacks of cardiopulmonary bypass and cardioplegic arrest in neonates needing open heart operations (McLennan et al.).

To summarize, hybrid surgery is an appealing therapeutic option for an increasing number of patients with congenital heart disease. At present, further studies are needed to clarify its feasibility on large scale and its short- and long-term clinical outcomes in different clinical scenarios. The cases presented in this Research Topic are very interesting and clinically relevant examples of how hybrid techniques can maximize benefits of both surgery and interventional cardiology while minimizing the risks, and certainly offering the chance for an unprecedented collaboration between cardiac surgeons, cardiologists, anesthetists, and radiologists.

## Author Contributions

Substantial contributions to the conception or design of the work; drafting the work or revising it critically for important intellectual content; final approval of the version to be published; and agreement to be accountable for all aspects of the work in ensuring that questions related to the accuracy or integrity of any part of the work are appropriately investigated and resolved: FR, DK, and MC.

## Disclaimer

This article/paper/report presents independent research funded by the National Institute for Health Research (NIHR). The views expressed are those of the author(s) and not necessarily those of the NHS, the NIHR, or the Department of Health.

## Conflict of Interest Statement

The authors declare that the research was conducted in the absence of any commercial or financial relationships that could be construed as a potential conflict of interest.
